# *In Vitro* Activity of Omadacycline, a New Tetracycline Analog, and Comparators against Clostridioides difficile

**DOI:** 10.1128/AAC.00522-20

**Published:** 2020-07-22

**Authors:** Khurshida Begum, Eugénie Bassères, Julie Miranda, Chris Lancaster, Anne J. Gonzales-Luna, Travis J. Carlson, Tasnuva Rashid, David W. Eyre, Mark H. Wilcox, M. Jahangir Alam, Kevin W. Garey

**Affiliations:** a University of Houston College of Pharmacy, Houston, Texas, USA; b Fred Wilson School of Pharmacy, High Point University, High Point, North Carolina, USA; c Big Data Institute, Nuffield Department of Population Health, University of Oxford, Oxford, United Kingdom; d National Institute for Health Research Oxford Biomedical Research Centre, Oxford, United Kingdom; e Healthcare Associated Infections Research Group, Leeds Institute for Medical Research, University of Leeds, Old Medical School, Leeds General Infirmary, Leeds, United Kingdom; f Microbiology, Leeds Teaching Hospitals NHS Trust, Old Medical School, Leeds General Infirmary, Leeds, United Kingdom

**Keywords:** anaerobe, pharmacology, susceptibility, time-kill studies

## Abstract

Omadacycline is a potent aminomethylcycline with *in vitro* activity against Gram-positive, Gram-negative, and anaerobic bacteria. Preliminary data demonstrated that omadacycline has *in vitro* activity against Clostridioides difficile; however, large-scale *in vitro* studies have not been done. The purpose of this study was to assess the *in vitro* susceptibility of omadacycline and comparators on a large biobank of clinical C. difficile isolates. *In vitro* C. difficile susceptibility to omadacycline and comparators (fidaxomicin, metronidazole, and vancomycin) was assessed using the broth microdilution method. Minimum bactericidal concentrations (MBCs) and time-kill assays were assessed for pharmacodynamics analysis, and whole-genome sequencing was performed in a subset of isolates to assess distribution of MICs and resistance determinants. Two hundred fifty clinical C. difficile isolates collected between 2015 and 2018 were tested for *in vitro* susceptibility of omadacycline and comparators. Ribotypes included F001 (*n* = 5), F002 (*n* = 56), F014-020 (*n* = 66), F017 (*n* = 8), F027 (*n* = 53), F106 (*n* = 45), and F255 (*n* = 17). Omadacycline demonstrated potent *in vitro* activity with an MIC range of 0.016 to 0.13 μg/ml, an MIC_50_ of 0.031 μg/ml, and an MIC_90_ of 0.031 μg/ml. No difference was observed for omadacycline MIC_50_ and MIC_90_ values stratified by ribotype, disease severity, or vancomycin susceptibility. Bactericidal activity was confirmed in time-kill studies. No difference was observed in MIC based on C. difficile phylogeny. Further development of omadacycline as an intravenous and oral antibiotic directed toward C. difficile infection is warranted.

## Abstract (precis)

Omadacycline is a potent aminomethylcycline with *in vitro* activity against Gram-positive, Gram-negative, and anaerobic bacteria. Preliminary data demonstrated that omadacycline has *in vitro* activity against Clostridioides difficile; however, large-scale *in vitro* studies have not been done. The purpose of this study was to assess the *in vitro* susceptibility of omadacycline and comparators on a large biobank of clinical C. difficile isolates.

## INTRODUCTION

Clostridioides difficile is a Gram-positive, anaerobic, spore-forming organism that produces two toxins, A and B, which represent the major virulence factors of the organism (1). C. difficile infection results primarily from the effects of these toxins on the intestine causing fluid accumulation, epithelial inflammation, diarrhea, pseudomembranous colitis, and death in severe cases (2–4). The spectrum of C. difficile infection symptoms ranges from diarrhea to life-threatening sepsis. Fulminant C. difficile infection is often characterized by ileus requiring intravenous (IV) therapy. Historically, metronidazole has been the IV drug of choice due to *in vitro* susceptibility and clinical experience. However, a major problem in the treatment of C. difficile infection has been the declining efficacy of metronidazole, especially for severe disease such that it is no longer recommended for nonfulminant C. difficile infection (5). Despite its weaknesses, metronidazole remains the treatment of choice if IV therapy is needed due to a lack of alternatives. Thus, there is an urgent unmet medical need to identify an IV antibiotic with *in vitro* and pharmacologic activity against C. difficile.

Tetracyclines are an antibiotic class at low risk for causing C. difficile infection. A study from San Francisco, CA, demonstrated decreased occurrence of C. difficile infection in patients with community-acquired pneumonia if their treatment regimen included doxycycline (6). In addition, tigecycline has been shown to decrease toxin production, inhibit spore formation, and demonstrated clinical efficacy used in patients with severe and fulminant C. difficile infection (7). However, tigecycline is associated with multiple toxicities that limit its use in clinical practice. Omadacycline is a potent aminomethylcycline with *in vitro* activity against Gram-positive, Gram-negative, and anaerobic bacteria (8). Omadacycline has recently completed phase 3 clinical trials for acute bacterial skin and skin structure infections and community-acquired bacterial pneumonia (9). Similar to other tetracyclines, omadacycline inhibits protein synthesis by binding to the 30S ribosomal subunit, although this antimicrobial has been structurally modified to overcome resistance, specifically via efflux mechanisms. Preliminary data demonstrated that omadacycline has *in vitro* and *in vivo* (animal model) efficacy against C. difficile (10, 11). However, a large-scale study to determine the *in vitro* activity of omadacycline has not been reported. The purpose of this study was to assess the *in vitro* susceptibility of omadacycline and comparators on contemporary, well-characterized clinical C. difficile isolates representing common ribotypes.

## RESULTS

### Minimum inhibitory activity of omadacycline against C. difficile.

Two hundred fifty clinical C. difficile isolates collected between 2015 and 2018 were tested for *in vitro* susceptibility of omadacycline and comparators. One hundred eighteen isolates (47%) were obtained from patients with mild-moderate disease, and 132 isolates (53%) were obtained from patients with severe disease. Ribotypes included F001 (*n* = 5), F002 (*n* = 56), F014-020 (*n* = 66), F017 (*n* = 8), F027 (*n* = 53), F106 (*n* = 45), and F255 (*n* = 17). Severe disease was more common for F017 (8 of 8 strains) and F027 (39 of 53 strains; 74%) than for 85 of 189 (45%) strains for the other ribotypes. Omadacycline demonstrated potent *in vitro* activity, with an MIC range of 0.016 to 0.13 μg/ml, an MIC_50_ of 0.031 μg/ml, and an MIC_90_ of 0.031 μg/ml. Metronidazole had an MIC range of 0.031 μg/ml to 4 with an MIC_50_ of 0.5 μg/ml and an MIC_90_ of 2 μg/ml. Vancomycin had a MIC range of 0.13 μg/ml to 4 with an MIC_50_ of 2 μg/ml and an MIC_90_ of 2 μg/ml. Fidaxomicin had a MIC range of 0.016 μg/ml to 0.25 with an MIC_50_ of 0.016 μg/ml and an MIC_90_ of 0.063 μg/ml. MIC determinations (MIC_50_, MIC_90_, and geometric mean MIC) by ribotype are shown in Table 1. No difference was observed for omadacycline MIC_50_ and MIC_90_ values stratified by ribotype. MIC_50/90_ values of omadacycline were within one 2-fold dilution for all ribotypes. Likewise, MIC values for omadacycline did not differ based on severity of disease presentation (Table 2) or vancomycin MIC (range, 0.016 to 0.063 μg/ml) (Table 3).

**TABLE 1:** MICs of omadacycline and comparator antibiotics against *C. difficile* (24 h MIC reading)

Ribotype (*n*)	Compound	MIC_50_	MIC_90_	Geometric mean MIC
Total (250)	Omadacycline	0.031	0.031	0.025
	Fidaxomicin	0.016	0.063	0.026
	Metronidazole	0.5	2	0.631
	Vancomycin	2	2	1.436
F001 (5)	Omadacycline	0.031	0.05	0.031
	Fidaxomicin	0.016	0.1	0.032
	Metronidazole	0.5	1	0.660
	Vancomycin	2	4	2.000
F002 (56)	Omadacycline	0.031	0.031	0.025
	Fidaxomicin	0.016	0.031	0.020
	Metronidazole	0.5	1	0.500
	Vancomycin	2	2	1.414
F014-020 (66)	Omadacycline	0.016	0.031	0.022
	Fidaxomicin	0.016	0.031	0.020
	Metronidazole	0.5	1	0.500
	Vancomycin	1	2	1.158
F017 (8)	Omadacycline	0.031	0.031	0.026
	Fidaxomicin	0.016	0.031	0.022
	Metronidazole	0.5	0.5	0.420
	Vancomycin	1	1	0.841
F027 (53)	Omadacycline	0.016	0.031	0.022
	Fidaxomicin	0.031	0.063	0.032
	Metronidazole	2	2	1.282
	Vancomycin	2	2	1.387
F106 (45)	Omadacycline	0.016	0.031	0.023
	Fidaxomicin	0.031	0.063	0.029
	Metronidazole	0.5	1	0.516
	Vancomycin	1	2	1.167
F255 (17)	Omadacycline	0.031	0.031	0.027
	Fidaxomicin	0.031	0.044	0.027
	Metronidazole	0.5	1	0.542
	Vancomycin	2	4	2.083

**TABLE 2:** Omadacycline MIC determined by C. difficile infection disease severity

Drug	MIC at mild-moderate severity (μg/ml) (*n* = 118)		MIC at high severity (μg/ml) (*n* = 132)	
	MIC_50_	MIC_90_	MIC_50_	MIC_90_
Omadacycline	0.016	0.031	0.031	0.031
Fidaxomicin	0.016	0.031	0.016	0.063
Metronidazole	0.5	1	0.5	2
Vancomycin	2	2	2	2

**TABLE 3:** Omadacycline MICs determined by vancomycin MICs

Vancomycin MIC (μg/ml)	Omadacycline MIC (μg/ml)	
	MIC_50_	MIC_90_
<1 (*n* = 43)	0.016	0.016
1 (*n* = 68)	0.016	0.031
2 (*n* = 126)	0.031	0.031
4 (*n* = 13)	0.031	0.063

**FIG 1:**
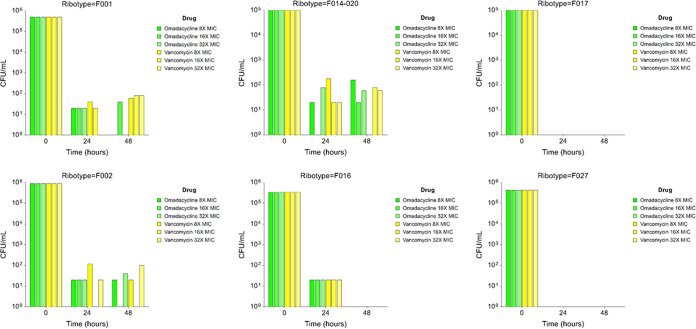
Time-kill experiments by ribotype, drug, and MIC.

The minimum bactericidal activity of omadacycline and vancomycin is shown in Table 4. MBCs for omadacycline were consistently lower than vancomycin for all ribotypes. MBC values ranged from 0.031 to 0.5 μg/ml for omadacycline and 0.5 to > 8 μg/ml for vancomycin. Time-kill studies demonstrated bactericidal activity at 24 and 48 h for omadacycline and vancomycin at 8×, 16×, and 32× the MIC of the organism (Fig. 1). MICs for omadacycline (0.031 μg/ml) and vancomycin (1.0 μg/ml) were the same for all isolates used in the time-kill studies.

**TABLE 4:** Minimum bactericidal activity of omadacycline and vancomycin against C. difficile clinical ribotypes (one isolate of each ribotype was tested)

Ribotype	Omadacycline		Vancomycin	
	MIC (μg/ml)	MBC (μg/ml)	MIC (μg/ml)	MBC (μg/ml)
F001	0.016	0.063	0.5	1
F002	0.015	0.063	0.5	8
F014-020	0.008	0.031	0.5	0.5
F016	0.016	0.063	1	4
F017	0.25	0.25	0.5	0.5
F027	0.125	0.5	>8.0	>8.0
F255	0.016	0.125	0.5	8

### Whole-genome sequencing and tet resistance determination.

**FIG 2:**
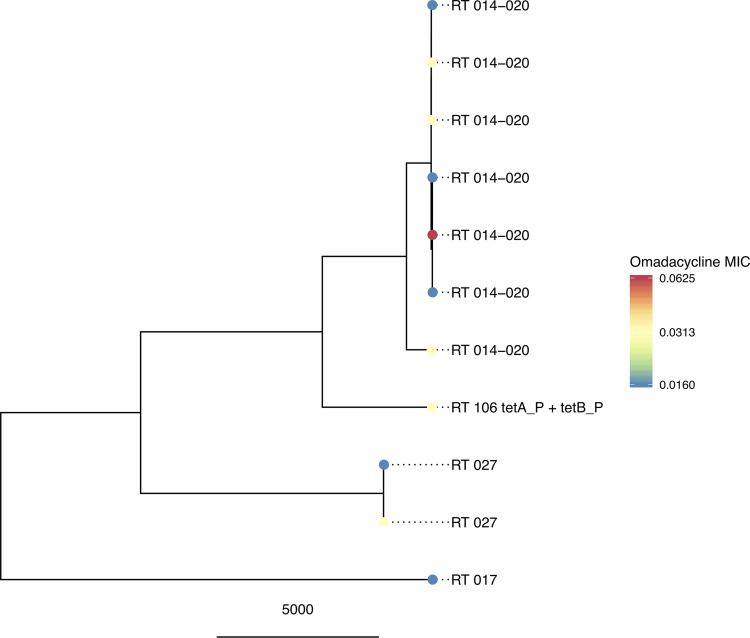
Phylogram of C. difficile isolates and tet resistance genes.

Sixteen isolates from ribotypes F014-020 (*n* = 8), F106 (*n* = 3), F017 (*n* = 1), F027 (*n* = 2), and F255 (*n* = 2) underwent whole-genome sequencing (Fig. 2). One F106 isolate was positive for the *tetA*(P) and *tetB*(P) resistance genes. MIC values ranged by three 2-fold dilutions (range, 0.016 to 0.063 μg/ml) and did not cluster by ribotype. The presence of the *tetA*/*tetB* resistance genes did not affect the omadacycline MIC of the isolate.

## DISCUSSION

C. difficile infection is the most common health care-associated infection in the United States, with an estimated 450,000 cases annually (12). Despite a high incidence of disease, treatment options are limited, especially for patients who require IV therapy. Metronidazole is the guideline-preferred IV antibiotic given there are no other viable intravenous options. However, metronidazole has been shown to be clinically inferior to vancomycin for C. difficile infection, and thus, an alternative IV option for C. difficile infection is needed. Omadacycline, an aminomethylcycline tetracycline antibiotic, has ideal characteristics of an IV antibiotic directed toward C. difficile infection in that it is primarily excreted unchanged in the feces (81%) and has been shown to not induce C. difficile infection in an *in vitro* gut model (10, 13).

This current study investigated the susceptibility of omadacycline and comparators against a large biobank of well-characterized C. difficile strains. In a previous investigation of 21 isolates, omadacycline MIC_50_ and MIC_90_ values were 0.25 and 0.5 μg/ml, respectively, using the agar dilution method for MIC determinations (11). In the current study, MIC_50_ and MIC_90_ values were 0.031 and 0.031 μg/ml, respectively, and did not differ based on ribotype, disease severity, or vancomycin MIC. While the MIC_50_ and MIC_90_ values were lower in this current study, many of our isolates had similar MIC values to the former study, which highlights the importance of evaluating MICs of novel antibiotics using a large collection of well-characterized strains. Although broth microdilution has been shown to produce reproducible results compared to agar dilution methods, broth microdilution can produce lower MIC values than agar dilution (14). Thus, differences in study methodology could also influence differences in these studies. One isolate with a *tet* resistance gene cluster was identified for which the omadacycline MIC did not differ compared to isolates of the same ribotype lacking a *tet* resistance gene cluster. These results are concordant with previous reports that suggest the *tetA* and *tetB* resistance genes have no effect on the MIC for omadacycline (15).

In previous studies with other organisms, omadacycline either displayed bacteriostatic activity (enterococci, Staphylococcus aureus, and Escherichia coli) or bactericidal activity (Streptococcus pneumoniae, Haemophilus influenzae, and Moraxella catarrhalis) (8). In the current study, MIC:MBC ratios were approximately 1:3 to 1:4 and did not differ based on ribotype. Time-kill curves also demonstrated bactericidal activity that was similar or more potent than comparator antibiotics tested. We chose six different ribotypes to perform the time-kill curves to better understand the pharmacologic effect of omadacycline over a diverse set of strains. However, future studies with a larger collection of isolates will be required to confirm these time-kill results. *In vitro* activity does not always correlate into *in vivo* activity, and further clinical studies will be necessary to determine if IV omadacycline may indeed be an alternative to metronidazole for treatment of fulminant C. difficile infection.

In conclusion, taking this current study and others into account, omadacycline was shown to have a low propensity of causing C. difficile infection in an *in vitro* model and confirmed in the subsequent clinical trials. It has pharmacokinetic properties favorable for a C. difficile infection antibiotic; namely, high rate of excretion of active antibiotic in the feces. This current study demonstrates potent *in vitro* activity of omadacycline against a contemporary collection of C. difficile isolates of a variety of ribotypes. Omadacycline should be considered an antibiotic at low risk of eliciting C. difficile infection when use is clinically indicated. Finally, further development of omadacycline as an intravenous and oral antibiotic directed toward C. difficile infection is warranted.

## MATERIALS AND METHODS

### Collection of isolates.

Isolates were obtained from our ongoing multicenter retrospective clinical study of patients with C. difficile infection hospitalized in two large health systems (13 hospitals in total) in the Houston, TX area (16). A medical chart review was performed for each patient in which an isolate was obtained to collect clinical meta-data, including C. difficile infection disease severity as defined by the 2017 Infectious Diseases Society of America (IDSA)/Society for Healthcare Epidemiology of America (SHEA) C. difficile guidelines (17). A randomly chosen, convenience sample of isolates obtained from 2017 to 2019 from patients ≥18 years of age with C. difficile infection who had specimen ribotype data available were selected for this study. The ongoing study is approved by the University of Houston Committee for the Protection of Human Subjects with a waiver of informed consent (IRB study 00000128).

### Microbiology and C. difficile identification.

Cryofrozen isolates were enriched overnight in brain heart infusion (BHI) broth (Criterion brain heart infusion broth; Hardy Diagnostics, Santa Maria, CA) supplemented with 0.05% sodium taurocholate and Oxyrase for broth (Oxyrase, Inc., Mansfield, OH) under anaerobic conditions. Overnight cultured isolates were streaked onto commercially prepared cycloserine cefoxitin fructose agar (CCFA) plates (Anaerobe Systems, Santa Clara, CA) and incubated at anaerobic conditions for 48 h. Isolates were confirmed to be C. difficile on the basis of Gram stain results and the presence of C. difficile antigen on Microscreen latex agglutination (Microgen Bioproducts Ltd., Surrey, United Kingdom). Fluorescent PCR ribotyping was performed as previously described (18, 19). For this study, clinical strains from the seven most common or emerging ribotypes in our collection corresponding to worldwide ribotypes using capillary gel ribotyping were used: F001, F002, F014-020, F017, F027, F106, and F255 (20).

### Antimicrobials.

Omadacycline was provided by the sponsor (Paratek Pharmaceuticals, Boston, MA). Metronidazole, fidaxomicin, and vancomycin were purchased by Sigma-Aldrich, Inc. (St. Louis, MO).

### *In vitro* susceptibility.

*In vitro* susceptibility of C. difficile to omadacycline and comparator antibiotics (fidaxomicin, metronidazole, and vancomycin) was assessed using the broth microdilution method as previously described (14). MIC panels containing 2-fold dilutions of omadacycline and comparators (range, 0.03 to 16 μg/ml) in supplemented BHI broth were prepared. Fidaxomicin was diluted in dimethyl sulfoxide (DMSO) and further diluted with distilled water to each final concentration. Each isolate was streaked onto a blood agar plate and incubated overnight. A single isolated colony from the blood agar plate was suspended in BHI/Mueller-Hinton (MH) broth to achieve turbidity equal to the 0.5 McFarland standard. One hundred microliters of the suspension were added to microtiter wells for a final concentration of ∼1 × 10^6^ CFU/ml. The MIC was defined as the lowest concentration of the agent that inhibited growth at 24 h. Reference strains (Bacteroides fragilis ATCC 25285, Bacteroides thetaiotaomicron ATCC 29741, and C. difficile ATCC 700057) were included as controls. All assays were performed at least in duplicate. The MIC was repeated for any results with disagreements with the duplicates.

### Minimum bactericidal assay.

One isolate from each ribotype was further assessed for MBC values. Following incubation and analysis of the MIC plates, 10-μl aliquots from the MIC well and three wells above the MIC were spotted onto the surface of prereduced Brucella agar supplemented with 5% sheep blood and vitamin K1 (1 mg/liter) to determine the MBC in accordance with Clinical and Laboratory Standards Institute (CLSI) guidelines (21). Plates were incubated anaerobically overnight at 37°C. The highest dilution that yielded no single colony was considered the MBC.

### Time-kill kinetic studies.

Cultures were prepared from one isolate of each C. difficile ribotype by inoculating 20 ml brain heart infusion-supplemented (BHIS) broth with a single colony of each ribotype. Cultures were grown for approximately 18 h to achieve turbidity equal to the 0.5 McFarland standard. One hundred microliters of the suspension was added to microtiter wells for a final concentration of ∼1 × 10^6^ CFU/ml. Concentration of omadacycline at 8×, 16×, or 32× the MIC was added along with negative controls. Total viable counts were determined immediately (T0) and at 24 and 48 h postinoculation. Samples were withdrawn at each time point, centrifuged (1 min at 16 ,000 × *g*), and washed twice in sterile prereduced phosphate-buffered saline (PBS) (Oxoid Ltd., Waltham, MA) to reduce residual drug carryover before 10-fold serial dilutions were performed prior to plating on BHIS agar. Agar plates were incubated for 24 h, following which the number of viable C. difficile (CFU/ml) was determined. The limit of detection (LOD) for killing kinetic assays was 50 CFU/ml. Bactericidal activity was defined as a reduction of ≥3 log_10_ in viability relative to the starting inoculum after 24 h exposure to antibiotics.

### Whole-genome sequencing and resistance gene determinants.

A convenience sample of 16 isolates from five distinct ribotypes underwent DNA extraction using either the QIAamp DNA minikit (Qiagen, Venlo, The Netherlands) or AnaPrep automated DNA extractor (BioChain Institute Inc., Newark, CA) as previously described (22). DNA was quantified by NanoDrop (Thermo Fisher Scientific, Waltham, MA) and Qubit (Thermo Fisher Scientific, Waltham, MA), and DNA quality was assessed using a BioAnalyzer (Agilent Technologies Inc., Santa Clara, CA). DNA libraries were prepared according to Illumina’s protocols, multiplexed on a flow cell, and run on a NextSeq (Illumina Inc., San Diego, CA) using paired-end sequencing. Sequence data were mapped against the 630 reference genomes as previously described (23). Sequences were compared using single-nucleotide polymorphisms (SNPs) obtaining differences between sequences from maximum-likelihood phylogenies constructed from mapped read data using PhyML version 3.1 (24) (with generalized time-reversible substitution model and BEST tree topology search algorithm) and corrected for recombination using ClonalFrameML version 1.25 (25) (with default settings). Sequence reads were also *de novo* assembled with Velvet (26) using the Velvet optimizer; BLAST searches were used to identify the presence of resistance genes, including *tetM*, *tetO*, *tetW*, *tetO/32/O*, *tetB*(P), *tet40*, *tetA*(P), *tetL* as in (27), and also *tetX* using an E value for screening for matches of 0.01. All matches were considered, including if spanning multiple contigs. Where present, all matches covered ≥95% of the respective *tet* genes.

## References

[1] Kuehne SA, Cartman ST, Heap JT, Kelly ML, Cockayne A, Minton NP 2010 The role of toxin A and toxin B in *Clostridium difficile* infection. Nature 467:711–713. doi:10.1038/nature09397. 20844489

[2] Carter GP, Rood JI, Lyras D 2010 The role of toxin A and toxin B in *Clostridium difficile*-associated disease: past and present perspectives. Gut Microbes 1:58–64. doi:10.4161/gmic.1.1.10768. 20664812 PMC2906822

[3] Warny M, Pepin J, Fang A, Killgore G, Thompson A, Brazier J, Frost E, McDonald LC 2005 Toxin production by an emerging strain of *Clostridium difficile* associated with outbreaks of severe disease in North America and Europe. Lancet 366:1079–1084. doi:10.1016/S0140-6736(05)67420-X. 16182895

[4] Voth DE, Ballard JD 2005 *Clostridium difficile* toxins: mechanism of action and role in disease. Clin Microbiol Rev 18:247–263. doi:10.1128/CMR.18.2.247-263.2005. 15831824 PMC1082799

[5] Johnson S, Louie TJ, Gerding DN, Cornely OA, Chasan-Taber S, Fitts D, Gelone SP, Broom C, Davidson DM, for the Polymer Alternative for CDI Treatment (PACT) investigators 2014 Vancomycin, metronidazole, or tolevamer for *Clostridium difficile* infection: results from two multinational, randomized, controlled trials. Clin Infect Dis 59:345–354. doi:10.1093/cid/ciu313. 24799326

[6] Doernberg SB, Winston LG, Deck DH, Chambers HF 2012 Does doxycycline protect against development of *Clostridium difficile* infection? Clin Infect Dis 55:615–620. doi:10.1093/cid/cis457. 22563022 PMC3491851

[7] Kechagias KS, Chorepsima S, Triarides NA, Falagas ME 2020 Tigecycline for the treatment of patients with Clostridium difficile infection: an update of the clinical evidence. Eur J Clin Microbiol Infect Dis 39:1053–1058. doi:10.1007/s10096-019-03756-z. 31927652

[8] Karlowsky JA, Steenbergen J, Zhanel GG 2019 Microbiology and preclinical review of omadacycline. Clin Infect Dis 69:S6–S15. doi:10.1093/cid/ciz395. 31367743 PMC6669291

[9] Lan SH, Chang SP, Lai CC, Lu LC, Chao CM 2019 The efficacy and safety of omadacycline in treatment of acute bacterial infection: a systemic review and meta-analysis of randomized controlled trials. Medicine (Baltimore, MD) 98:e18426. doi:10.1097/MD.0000000000018426. PMC6940113 31861009

[10] Moura IB, Buckley AM, Ewin D, Shearman S, Clark E, Wilcox MH, Chilton CH 2018 Omadacycline gut microbiome exposure does not induce *Clostridium difficile* proliferation or toxin production in a model that simulates the proximal, medial, and distal human colon. Antimicrob Agents Chemother 63:e01581-18. doi:10.1128/AAC.01581-18. PMC6355569 30455242

[11] Stapert L, Wolfe C, Shinabarger D, Marra A, Pillar C 2018 In vitro activities of omadacycline and comparators against anaerobic bacteria. Antimicrob Agents Chemother 62:e00047-18. doi:10.1128/AAC.00047-18. 29439961 PMC5913939

[12] Lessa FC, Mu Y, Bamberg WM, Beldavs ZG, Dumyati GK, Dunn JR, Farley MM, Holzbauer SM, Meek JI, Phipps EC, Wilson LE, Winston LG, Cohen JA, Limbago BM, Fridkin SK, Gerding DN, McDonald LC 2015 Burden of *Clostridium difficile* infection in the United States. N Engl J Med 372:825–834. doi:10.1056/NEJMoa1408913. 25714160 PMC10966662

[13] Rodvold KA, Pai MP 2019 Pharmacokinetics and pharmacodynamics of oral and intravenous omadacycline. Clin Infect Dis 69:S16–S22. doi:10.1093/cid/ciz309. 31367744 PMC6669312

[14] Citron DM, Goldstein EJ 2011 Reproducibility of broth microdilution and comparison to agar dilution for testing CB-183,315 against clinical isolates of *Clostridium difficile*. Diagn Microbiol Infect Dis 70:554–556. doi:10.1016/j.diagmicrobio.2011.04.012. 21767714

[15] Fluit AC, van Gorkum S, Vlooswijk J 2019 Minimal inhibitory concentration of omadacycline and doxycycline against bacterial isolates with known tetracycline resistance determinants. Diagn Microbiol Infect Dis 94:78–80. doi:10.1016/j.diagmicrobio.2018.11.010. 30583881

[16] Carlson TJ, Endres BT, Le Pham J, Gonzales-Luna AJ, Alnezary FS, Nebo K, Miranda J, Lancaster C, Basseres E, Begum K, Alam MJ, Reveles KR, Garey KW 2020 Eosinopenia and binary toxin increase mortality in hospitalized patients with *Clostridioides difficile* infection. Open Forum Infect Dis 7:ofz552. doi:10.1093/ofid/ofz552. 31993458 PMC6979314

[17] McDonald LC, Gerding DN, Johnson S, Bakken JS, Carroll KC, Coffin SE, Dubberke ER, Garey KW, Gould CV, Kelly C, Loo V, Shaklee Sammons J, Sandora TJ, Wilcox MH 2018 Clinical practice guidelines for *Clostridium difficile* infection in adults and children: 2017 update by the Infectious Diseases Society of America (IDSA) and Society for Healthcare Epidemiology of America (SHEA). Clin Infect Dis 66:987–994. doi:10.1093/cid/ciy149. 29562266

[18] Martinson JN, Broadaway S, Lohman E, Johnson C, Alam MJ, Khaleduzzaman M, Garey KW, Schlackman J, Young VB, Santhosh K, Rao K, Lyons RH Jr, Walk ST 2015 Evaluation of portability and cost of a fluorescent PCR ribotyping protocol for *Clostridium difficile* epidemiology. J Clin Microbiol 53:1192–1197. doi:10.1128/JCM.03591-14. 25631804 PMC4365229

[19] Alam MJ, Anu A, Walk ST, Garey KW 2014 Investigation of potentially pathogenic *Clostridium difficile* contamination in household environs. Anaerobe 27:31–33. doi:10.1016/j.anaerobe.2014.03.002. 24657158

[20] Gonzales-Luna AJ, Carlson TJ, Dotson KM, Poblete K, Costa G, Miranda J, Lancaster C, Walk ST, Tupy S, Begum K, Alam MJ, Garey KW 2020 PCR ribotypes of *Clostridioides difficile* across Texas from 2011 to 2018 including emergence of ribotype 255. Emerg Microbes Infect 9:341–347. doi:10.1080/22221751.2020.1721335. 32037964 PMC7033716

[21] Clinical and Laboratory Standards Institute. 2014 Performance standards for antimicrobial susceptibility testing: 24th informational supplement. CLSI M100-S24 Clinical and Laboratory Standards Institute, Wayne, PA.

[22] Endres BT, Begum K, Sun H, Walk ST, Memariani A, Lancaster C, Gonzales-Luna AJ, Dotson KM, Basseres E, Offiong C, Tupy S, Kuper K, Septimus E, Arafat R, Alam MJ, Zhao Z, Hurdle JG, Savidge TC, Garey KW 2019 Epidemic *Clostridioides difficile* ribotype 027 lineages: comparisons of Texas versus worldwide strains. Open Forum Infect Dis 6:ofz013. doi:10.1093/ofid/ofz013. 30793006 PMC6368847

[23] Eyre DW, Cule ML, Wilson DJ, Griffiths D, Vaughan A, O'Connor L, Ip CLC, Golubchik T, Batty EM, Finney JM, Wyllie DH, Didelot X, Piazza P, Bowden R, Dingle KE, Harding RM, Crook DW, Wilcox MH, Peto TEA, Walker AS 2013 Diverse sources of *C. difficile* infection identified on whole-genome sequencing. N Engl J Med 369:1195–1205. doi:10.1056/NEJMoa1216064. 24066741 PMC3868928

[24] Guindon S, Gascuel O 2003 A simple, fast, and accurate algorithm to estimate large phylogenies by maximum likelihood. Syst Biol 52:696–704. doi:10.1080/10635150390235520. 14530136

[25] Didelot X, Wilson DJ 2015 ClonalFrameML: efficient inference of recombination in whole bacterial genomes. PLoS Comput Biol 11:e1004041. doi:10.1371/journal.pcbi.1004041. 25675341 PMC4326465

[26] Zerbino DR, Birney E 2008 Velvet: algorithms for de novo short read assembly using de Bruijn graphs. Genome Res 18:821–829. doi:10.1101/gr.074492.107. 18349386 PMC2336801

[27] Dingle KE, Didelot X, Quan TP, Eyre DW, Stoesser N, Marwick CA, Coia J, Brown D, Buchanan S, Ijaz UZ, Goswami C, Douce G, Fawley WN, Wilcox MH, Peto TEA, Walker AS, Crook DW 2019 A role for tetracycline selection in recent evolution of agriculture-associated C*lostridium difficile* PCR ribotype 078. mBio 10:e02790-18. doi:10.1128/mBio.02790-18. PMC6414706 30862754

